# Diagnosis of smear-negative tuberculosis is greatly improved by Xpert MTB/RIF

**DOI:** 10.1371/journal.pone.0176186

**Published:** 2017-04-21

**Authors:** Giulia Lombardi, Valentina Di Gregori, Nicolò Girometti, Marina Tadolini, Francesco Bisognin, Paola Dal Monte

**Affiliations:** 1 Department of Experimental, Diagnostic and Specialty Medicine - Unit of Microbiology, Alma Mater Studiorum University of Bologna - S. Orsola-Malpighi University Hospital, Bologna, Italy; 2 Department of Biomedical and Neuromotor Sciences, Section of Hygiene and Public Health, Alma Mater Studiorum University of Bologna, Bologna, Italy; 3 Department of Medical and Surgical Sciences - Unit of Infectious Diseases, Alma Mater Studiorum University of Bologna - S. Orsola-Malpighi University Hospital, Bologna, Italy; University College London, UNITED KINGDOM

## Abstract

**Background:**

Diagnosis of pulmonary (PTB) and extra-pulmonary tuberculosis (EPTB) in smear-negative patients can be difficult. We assessed retrospectively the performance of Xpert MTB/RIF system (Xpert, Cepheid) in diagnosing smear-negative tuberculosis (TB), which represents the most common form of TB in a low incidence setting.

**Methods:**

Performance of Xpert was compared to acid-fast microscopic examination using Ziehl-Neelsen (ZN) stain in patients with culture-confirmed TB.

**Results:**

386 *Mycobacterium tuberculosis* (MTB) culture-positive samples were detected out of 5170 specimens tested with smear microscopy, Xpert and culture: 323 were both culture- and Xpert-positive, and 63 culture-positive only. Of these, 234 (60.6%) were smear-negative. In addition Xpert detected 40 probable TB cases, based on clinical findings, which were culture-negative.

Compared to culture, Xpert showed an overall sensitivity of 83.7% and a specificity of 99.1%; sensitivity was higher for respiratory samples (86.5%) than for non-respiratory samples (76.8%). Xpert sensitivity for smear-negative culture-confirmed TB was 73.1% and was not influenced by TB localization. As sensitivity of microscopy alone was poor (39.4%), Xpert improved both diagnosis of pulmonary TB (Δ = 36.5%) and extra-pulmonary TB (Δ = 63.4%).

**Conclusions:**

Xpert MTB/RIF is a sensitive method for rapid diagnosis of TB compared to the conventional ZN staining. Xpert can serve as a sensitive and time-saving diagnostic method for microbiological diagnosis of smear-negative TB in countries with a low TB prevalence.

## Introduction

The diagnosis of tuberculosis (TB) still offers big diagnostic challenges related to the detection limit of smear microscopy, long time to culture-confirmation and variable sensitivity of molecular tests.

In particular, diagnosing active smear-negative pulmonary TB (PTB), which represent the majority of TB cases is a major concern. According to the 2015 European Centre for Disease Prevention (ECDC) report, out of all PTB cases diagnosed in Italy in 2014, 68.1% were smear-negative [1]. Diagnostic delays and poor microbiological accuracy within these cases lead to a late response to clinicians and consequently to a delayed optimal treatment and poorer treatment response [2].

Also, the diagnosis of extra-pulmonary tuberculosis (EPTB) still represents a challenge, due to its paucibacillary nature leading to a prolonged time before the diagnosis occur. The number of EPTB is quite high in industrialized countries such as Italy, where it represents the 22.4% of total TB cases notified [1]. Improving the diagnostic accuracy and reducing diagnostic delay in both smear-negative PTB and EPTB is therefore paramount.

Xpert MTB/RIF (Xpert; Cepheid, USA) is a fully automated real time hemi-nested PCR system which simultaneously detects *Mycobacterium tuberculosis* complex (MTB) genome as well as mutations that confer rifampicin resistance. It has been recently endorsed by the Scientific and Technical Advisory Board of the World Health Organization as the most sensitive rapid test for TB diagnosis in paucibacillary respiratory samples [3]. This on-demand assay requires less than 2 hours to be performed and incorporate the steps of bacterial lysis, DNA extraction, amplification and amplicon detection using a disposable plastic cartridge thus acting as a “lab-on-chip” device which run on the GeneXpert platform [4]. The advantages of this test include high sensitivity and specificity, low complexity, a minimum hands-on time and negligible safety concerns for persons handling the samples [5–9]. Many data have been published with the aim to define whether Xpert could improve the diagnostic process in low-incidence countries, with conflicting findings. A study performed in Canada suggested a limited potential impact of Xpert due to low sensitivity of the assay in the context of less extensive disease in high-resource, low-incidence settings (overall sensitivity 46%; sensitivity in smear-negative 29%) [10]. Otherwise, Opota and colleagues found out an high sensitivity of Xpert (91.5%) in pulmonary TB in a similar setting [11].

In this study we aimed to assess the performance of the Xpert MTB/RIF system on a large number of respiratory and non-respiratory samples in a low incidence high-resource setting, with particular attention to the confirmation of TB in smear-negative cases.

## Materials and methods

### Study design

This is a 5-year (January 2011-December 2015) retrospective analysis of microbiological results for the diagnosis of TB performed at the Unit of Microbiology of the S. Orsola-Malpighi University Hospital in Bologna (Italy), a city in Emilia-Romagna Region (Northern Italy), where TB notification rate was 12.1 cases/100,000 inhabitants in 2011, with a high proportion of foreign-born patients (67.5%) [12,13].

The study was approved by the Ethics Committee of S.Orsola-Malpighi University Hospital (Approval number 1104/2016). The patients’ written informed consent to participate to this study was always obtained whenever possible and filed in their medical records; in case patients were no longer traceable their data have been utilized as agreed by Ethics Committee in line with regulation n 72 of 26 March 2012 issued by Privacy Warrantor [14].

During the study period, 5170 specimens originated from patients with suspected TB have been examined with Xpert MTB/RIF. On all samples, smear microscopy and mycobacterial culture have been performed too.

### Microbiological diagnosis of TB

#### Smear microscopy

All specimens were stained for acid-fast microscopic examination using Ziehl-Neelsen stain, before sample concentration. The grade of acid fast bacilli positivity was assigned to one of the four categories (1+, 2+, 3+, 4+) as per Clinical and Laboratory Standards Institute (CLSI) guidelines [15, 16].

#### Culture

After decontamination with N-Acetyl-L-Cysteine-Sodium Hydroxide (NALC-NaOH) method (MycoPrep, Becton Dickinson, USA), specimens were inoculated in solid (Lowenstein-Jensen; Heipha Diagnostika Biotest, Germany) and liquid culture (MGIT 960; Becton Dickinson, USA). Solid and liquid cultures were considered negative after 42 days of incubation without isolation of any Mycobacteria.

#### Identification of mycobacteria

Positive culture were identified as MTB by MGIT TBc Identification Test (Becton Dickinson, USA), or as Non-Tuberculous Mycobacteria (NTM) by Genotype CM (Hain, Germany). Drug susceptibility test (DST) to first-line drugs (Streptomycin, Isoniazid, Rifampicin, Ethambutol, Pyrazinamide) of MTB isolates was performed by the “gold standard” automatic MGIT 960 system (Becton Dickinson).

#### Xpert procedure

Xpert assay was performed following the producer’s suggested protocol. Tests conducted between January 2011 and April 2012 have been performed with G3 assay version and thereafter G4 version. Briefly, 500 μl of decontaminated and concentrated sample was pre-treated with a Sample Solution (containing NaOH and isopropanol) at a 1:3 ratio for 15 minutes at room temperature and poured into a single-use disposable cartridge of the GeneXpert module [17]. The system automatically interpreted all results from measured fluorescent signal into the following categories: invalid, if PCR inhibitors were detected with amplification failure, negative or positive. Positive results were scaled into 4 categories (very low, low, medium, high) depending on bacterial load and defined susceptible or resistant to rifampicin depending on detection of mutations in *rpoB* gene.

### Definition of TB cases

In this study we considered confirmed TB cases all those with positive MTB culture. For patients whose samples were Xpert-positive but culture-negative, probable TB was diagnosed based on clinical and radiological findings, previous and/or current history of TB treatment and treatment response.

### Statistical analysis

Determination of sensitivity, specificity and their CI at 95% level of significance was performed using MedCalc tool available on line (https://www.medcalc.org/calc/diagnostic_test.php). Comparison between categorical variables was made using Fisher’s test; a p value lower than 0.05 was considered statistically significant.

Percentage change (Δ%) was computed from the raw disaggregated data and then exact binomial confidence interval for proportion in small sample with 95% confidence interval was performed. All these analyses were computed using the statistical software Stata/SE 12.1 (StataCorp^®^, USA).

## Results

### Xpert results

**Fig 1:**
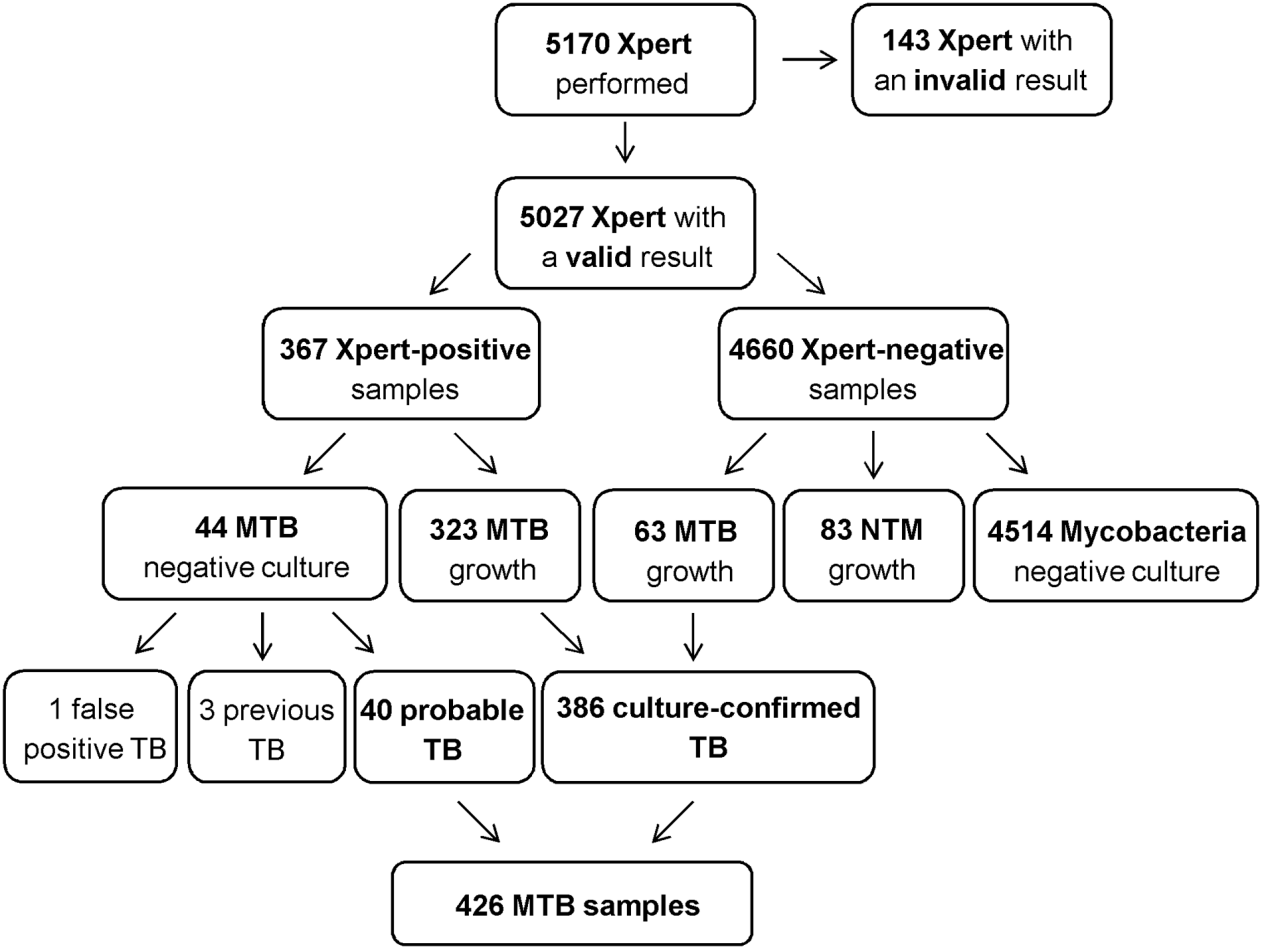
Flow-chart of the study. MTB: *Mycobacterium tuberculosis*, NTM: Non-Tuberculous Mycobacteria.

The flow-chart of the study is reported in Fig 1: out of 5170 Xpert tests performed (883 with G3 assay version and 4287 with G4 version), 143 (2.8%) had an invalid result and were excluded from the analysis. Similar proportion of invalid results were obtained with the two assay versions (2.3% with G3 and 2.9% with G4). Among 5027 samples with a valid result, 3067 were respiratory (sputum, broncoalveolar lavage and gastric aspirates), and 1960 non-respiratory, such as cavitary fluids, lymphnode tissue, bone samples, cerebrospinal fluid, other organs bioptic material, pus, urine.

Xpert resulted positive in 367 (7.3%) and negative in 4660 (92.7%) specimens. Among Xpert-negative samples, culture yielded 63 MTB and 83 NTM growths, respectively. Among Xpert-positive samples, culture positive for MTB was yielded in 323 (88.0%). Out of 44 Xpert-positive/culture-negative samples, 40 samples were from probable TB cases based on clinical and radiological findings and response to anti-TB therapy, while 3 were from previously treated TB cases and 1 was a false positive without clinical TB findings. Therefore, a total of 426 MTB samples (386 culture-confirmed and 40 probable TB) were considered. Out of them, Xpert resulted positive in 261/298 (87.6%) respiratory and in 102/128 (79.7%) non-respiratory samples. Among 298 respiratory samples, 105/114 (92.1%) sputum samples, 148/169 (87.6%) broncoalveolar lavage specimens and 8/15 (53.3%) gastric aspirates were Xpert-positive. Among 128 non-respiratory MTB samples, 50/56 (89.3%) lymphnode specimens, 18/23 (78.3%) bone samples, 12/16 (75.0%) pus, 9/12 (75.0%) cavitary fluids, 7/14 (50.0%) bioptic materials and 6/7 (85.7%) urine samples were Xpert-positive.

Among 426 MTB samples, 153 were smear-positive (35.9%) while 273 (64.1%) were smear-negative. As expected, smear-negative were predominantly obtained from extra-pulmonary sites (112/128 = 87.5%) when compared to pulmonary localization (161/298 = 54.0%) (p<0.001) (S1 Table).

All 153 smear-positive MTB samples were Xpert-positive, with the following grading: very low in 6, low in 23, medium in 78 and high in 46 cases. Among 273 smear-negative MTB samples, Xpert resulted negative in 63 cases, positive with very low grading in 83, low in 110, medium in 14 and high in 3 cases. Distribution of quantitative Xpert results in smear-negative samples did not significantly differ between respiratory and non-respiratory samples (p = 0.727).

Xpert detected 17 Rifampicin resistant cases, 15 also confirmed by phenotypic DST performed by the automatic MGIT 960 with a 100% concordance for Rifampicin resistance detection; conversely, two Rifampicin-Resistant-TB cases were detected only by Xpert as culture was negative.

### Xpert sensitivity and specificity

Table 1 reports Xpert sensitivity and specificity in MTB culture-positive samples. Xpert showed an overall sensitivity of 83.7% (323/386), higher for respiratory samples (237/274, 86.5%) than for non-respiratory samples (86/112, 76.8%). Sensitivity of G3 and G4 versions was 84.5% and 83.4% respectively.

**Table 1:** Smear sensitivity, Xpert sensitivity and specificity, added value (Δ%) of Xpert compared to smear microscopy in MTB culture-positive samples.

	Sample, n	MTB culture-positive, n	Smear Sensitivity, % (95% CI)	Xpert Sensitivity, % (95% CI)	Δ Xpert vs Smear, % (95% CI)	Xpert Specificity, % (95% CI)
**Total**	5027	386	39.4 (34.5–44.5)	83.7 (79.6–87.2)	44.3 (39.4–49.2)	99.1 (98.7–99.3)
**By sample type**						
Respiratory	3067	274	50.0 (43.9–56.1)	86.5 (81.9–90.3)	36.5 (30.8–42.2)	99.0 (98.6–99.4)
Non- respiratory	1960	112	13.4 (7.7–21.1)	76.8 (67.9–84.2)	63.4 (54.5–72.3)	99.1 (98.5–99.5)
**By assay version**						
G3	863	84		84.5 (75.0–91.5)		98.8 (97.8–99.5)
G4	4164	302		83.4 (78.8–87.5)		99.1 (98.7–99.4)
**In smear-negative**						
Total	4860	234		73.1 (66.9–78.7)		99.1 (98.8–99.3)
Respiratory	2917	137		73.0 (64.8–80.2)		99.0 (98.6–99.4)
Non-respiratory	1943	97		73.2 (63.2–81.7)		99.1 (98.6–99.5)

Overall sensitivity of smear microscopy in culture-positive samples was poor (152/386, 39.4%), slightly higher in PTB (137/274, 50.0%) than EPTB (15/112, 13.4%).

Xpert sensitivity in smear-positive samples was 100%. Xpert contributed to identify 171 additional smear-negative culture-positive specimens. Therefore, Xpert increased TB detection by 44.3% compared to smear microscopy; Xpert added value was more evident in EPTB (Δ%EPTB = 63.4%) than PTB (Δ%PTB = 36.5%).

Out of 234 smear-negative culture-positive samples, 137 (58.5%) were respiratory and 97 (41.5%) non-respiratory. Sensitivity of Xpert in smear-negative culture-confirmed TB was 73.1% (171/234): 73.0% (100/137) for PTB and 73.2% (71/97) for EPTB, respectively. Overall specificity of Xpert was 99.1% with no differences based on sample type and assay version. Similar specificity was achieved in smear-negative samples.

## Discussion

Since its introduction on the market, many data have been published on Xpert performance in diagnosing TB [18], especially in high-incidence countries, where the number of TB cases diagnosed per year increased by 30–37% due to its high sensitivity (100% in smear-positive and 79% in smear-negative cases), with a consequent improvement of the diagnostic process and related cost savings [19].

However, data defining whether Xpert could improve the diagnostic process in low-incidence countries where the majority of clinical specimens are smear-negative, such as Italy, are scarce and consisting in few reports only [1].

In our study performed on a large number of samples, Xpert showed a sensitivity of 83.7% compared to culture, which is in line with results published by Opota et al [11] and higher compared to results obtained in similar low incidence setting by Sohn et al [10]. The high sensitivity achieved in our study could be due to the use of Xpert on concentrated samples.

Xpert showed a high sensitivity also in non-respiratory samples (76.8%), obtaining the best performances on lymphnode (sensitivity 89.3%), urines (85.7%), and bone samples (78.3%), while it underperformed in cavitary fluids (50.0%), as previously reported [20, 21].

As expected, most (60.6%) of MTB culture-confirmed samples in our study were smear-negative, especially the non-respiratory samples.

Compared to culture, the Xpert sensitivity in smear-negative samples was 73.1% and was independent from the specimen origin. Studies evaluating Xpert performance in smear-negative pulmonary TB showed a sensitivity ranging from 47% to 87% [22–27], and around 66% in non-respiratory samples [28]. Our data score high compared to these studies.

Overall Xpert sensitivity was found not to be influenced by TB prevalence, but by smear status as described in a large meta-analysis performed with the Cochrane meta-analysis [29] and in a recent study by Luetkemeyer et al [30]. Our data support these findings, as performance of Xpert was limited by the high proportion of smear-negative samples.

Xpert allowed early MTB detection in 100 PTB and 71 EPTB smear-negative samples, increasing PTB case detection by 36.5% and EPTB by 63.4% compared to microscopy and enabling earlier appropriate treatment initiation. Shortening time to treatment of smear-negative patients, whose diagnosis would be delayed until culture results are available, may improve treatment response and save health resources. In a country with a similar low TB burden, Munoz and colleagues reported a sensitivity for smear-negative samples of 68%, suggesting that Xpert may offer a useful and cost-effective way to diagnose smear-negative pulmonary TB [31].

Interestingly, Xpert detected 44 samples, which were culture-negative. Out of them, 40 belonged to patients with probable TB (24 respiratory, 16 non-respiratory). For them discrepant Xpert positive-culture negative results may be ascribed to ongoing anti-TB treatment in 14 cases, and previous anti-TB treatment in 7 cases. The remaining 19 cases did not have a previous history of TB but showed good treatment response. The Xpert added value in diagnosing TB even when no supporting bacteriological evidence is available was also demonstrated by Wei et al, who analyzed many specimens collected from bone and joint tuberculosis suspects [32].

Rifampin resistance was uncommon in our study (4.1%), however Xpert demonstrated 100% concordance with DST and detected two further cases which were culture-negative. As conventional DST is slow and cumbersome, Xpert overcomes this delay leading to the enrolment on appropriate treatment to the patient, thus reducing the risk of spread and poor outcome.

As previously reported [11,33], we observed a correlation between smear grading and Xpert quantitative results. However, the high proportion (49.3%) of smear-negative Xpert-positive results could be partially ascribed to performing smear microscopy directly on unprocessed sample, differently from Xpert that was carried out on concentrated sample. In line with this evidence, recently, Tadesse and colleagues demonstrated that bleach concentration and pelleting of smear-negative samples increase Xpert sensitivity from 63.2% to 73.8% [34].

This study has some limitations. Firstly, overall sensitivity of microscopy was poor (39.4%), probably due to Ziehl-Neelsen staining before sample concentration. Nevertheless other studies in the same epidemiological setting reported similar sensitivity for smear microscopy. In a recent French study, Xpert assay significantly outperformed smear microscopy on fibreoptic bronchoscopy sampling for TB diagnosing (sensitivities: 80% vs 25%, p = 0.003) [35].

Secondly, the small number of paediatric, MDR-TB and co-infected HIV/TB patients could not lead to include statistical analysis of these subgroups.

In conclusion, our study confirms the clinical utility of Xpert in diagnosing TB, especially in smear-negative cases, both in PTB and EPTB, shortening the time to treatment.

## Supporting information

S1 Table Smear, culture and Xpert results of MTB samples according to their localization. (DOC)
