# Supplementary material for: Diagnosis of smear-negative tuberculosis is greatly improved by Xpert MTB/RIF
Source: PLoS One. 2017 Apr 21;12(4):e0176186. doi: 10.1371/journal.pone.0176186 (PMC5400262; doi:10.1371/journal.pone.0176186)
Supplement: S1 Table — (DOC) [file pone.0176186.s001.doc]

|  |  |  | **Smear-negative MTB samples**  **n=273** | | | | **Smear-positive MTB samples**  **n=153** | | | |
| --- | --- | --- | --- | --- | --- | --- | --- | --- | --- | --- |
|  |  |  | **Xpert Positive**  **n=210** | | **Xpert Negative**  **n=63** | | **Xpert Positive**  **n=153** | | **Xpert Negative**  **n=0** | |
| **Samples** | **Total Xpert,**  **n (%)** | **Total MTB**  **samples,**  **n (%)** | **MTB Culture Positive** | **MTB Culture Negative** | **MTB Culture Positive** | **MTB Culture Negative** | **MTB Culture Positive** | **MTB Culture Negative** | **MTB Culture**  **Positive** | **MTB Culture**  **Negative** |
| **All** | 5027 | 426 | 171 | 39 | 63 | 0 | 152 | 1 | 0 | 0 |
| **Respiratory** | 3067 (61) | 298  (70) | 100 | 24 | 37 | 0 | 137 | 0 | 0 | 0 |
| **Non-respiratory** | 1960 (39) | 128  (30) | 71 | 15 | 26 | 0 | 15 | 1 | 0 | 0 |

**S1 Table**
